# Circulating soluble fibroblast activation protein (FAP) in patients with intra-thoracic cancer undergoing chest radiation

**DOI:** 10.1007/s12672-025-02998-y

**Published:** 2025-07-21

**Authors:** Tim Lange, Alexandra Renko, Ulrike Flierl, Felix B. Ademmer, Johann Bauersachs, Hans Christiansen, Jochen Tillmanns

**Affiliations:** 1 Department of Radiation Oncology, Hannover Medical School, Carl-Neuberg-Str. 1, 30625 Hannover, Germany; 2 Department of Cardiology and Angiology, Hannover Medical School, Carl-Neuberg-Str. 1, 30625 Hannover, Germany

**Keywords:** Fibroblast activation protein α, Radiation injury, Activated fibroblasts, Circulating FAP

## Abstract

**Introduction:**

Circulating soluble fibroblast activation protein (FAP) is implicated in myocardial infarction, stroke, fibrosis and various cancers. This study investigates changes in FAP concentrations in patients with intra-thoracic malignancies undergoing chest radiation therapy to assess its potential as a biomarker for radiation-induced organ injury.

**Methods:**

Eighteen patients with intra-thoracic cancers (lung, esophagus and metastatic) received chest radiation therapy. Blood samples were taken before and after treatment, and FAP concentrations were measured using an ELISA assay. A control group of 53 healthy volunteers was included for comparison.

**Results:**

Baseline FAP concentrations were significantly lower in cancer patients (median 91 ng/mL, 25th-75th percentiles 72–123 ng/mL) compared to healthy controls (median 118 ng/mL, 25th-75th percentiles 104–140 ng/mL, *P* = 0.0002). No significant difference in FAP concentrations was found between baseline and post-radiation samples (median 91 vs. 108 ng/mL, *P* = 0.19). FAP concentrations were not influenced by cancer type, radiation dose or chemotherapy and did not predict patient survival. Time between baseline and final blood sampling was 31 days median (range 9–46 days) and median follow-up period was 20 months (range 15–30 months).

**Conclusion:**

Our findings indicate that FAP concentrations do not reflect radiation-induced inflammation or fibrosis in the early period after radiation therapy. The small patient cohort and short duration between radiation therapy and FAP measurement may have limited our ability to detect changes in FAP related to long-term radiation effects. Further research with larger cohorts and longer follow-up periods is needed to better understand the role of FAP in cancer and response to radiation injury.

**Supplementary Information:**

The online version contains supplementary material available at 10.1007/s12672-025-02998-y.

## Introduction

Chest radiation therapy is clinically applied for treatment of various cancers including lung and esophageal tumors [1, 2]. However, radiation-induced inflammation can lead to fibrosis, potentially affecting the lungs, heart, and esophagus [3, 4]. After stereotactic radiation for lung cancer, up to 50% of patients demonstrated pneumonitis and 14% of patients esophagitis indicating acute inflammatory response [3]. Experimentally, TGFβ_1_ has been identified as a key cytokine mediating this acute injury [5].

Early detection of radiation-induced inflammation and fibrosis is crucial for timely treatment and mitigation of adverse effects in patients undergoing chest radiation. Several biomarkers, including microRNAs and surface-related proteins, have been explored for this purpose [6, 7]. However, the sensitivity and specificity of these biomarkers are not yet sufficient for routine clinical use. In this regard, soluble fibroblast activation protein α (FAP), which has been implicated in various fibrotic and malignant diseases, may provide insight into radiation-induced tissue remodeling, although its utility as an early detection biomarker after radiation has not been studied.

Fibroblast activation protein α (FAP) is a serine protease expressed by activated fibroblasts in cardiovascular disease such as acute myocardial infarction and aortic stenosis [8–10] as well as many cancers [11–14]. Increased expression of FAP has been also described in fibrotic diseases such as idiopathic pulmonary fibrosis [15] and liver cirrhosis [16]. FAP is expressed by a TGFβ-driven mechanism in cardiac fibroblasts [8] and cancer associated fibroblasts [17], promoting fibroblast migration and exerting gelatinolytic activity in vitro [8]. Of note, FAP expression is absent or very low in healthy tissues [11, 18].

A soluble form of FAP has been detected in blood [19], and reduced blood concentrations in acute MI, stroke or malignancy were observed [14, 20–22]. However, circulating FAP has not been studied in patients undergoing radiation therapy and its potential effects on circulating FAP concentrations are unknown. We therefore hypothesized that chest radiation may alter circulating FAP concentrations in patients with intra-thoracic cancer.

## Materials and methods

### FAP ELISA

FAP concentrations in blood plasma samples were quantified using a commercial ELISA (R&D Systems) according to manufacturer instructions and as described previously [10].

Briefly, 96 well EIA plates were coated with mouse anti-human FAP antibody in phosphate buffered saline overnight at room temperature and washed thrice with PBS containing 0.05% Tween-20. Blocking was performed using reagent diluent consisting of 1% BSA in PBS for 1 h at room temperature followed by another washing step. Blood plasma (diluted 1:100) was added to the wells. After a washing step, 100 µL of 200 ng/mL biotinylated polyclonal sheep anti-human FAP antibody in reagent diluent was added to each well. The plate was then covered with a new adhesive strip and incubated for 2 h at room temperature. After a washing step, streptavidin-HRP was added to each well and incubated for 20 min at room temperature. After a washing step, 100 µL substrate solution was added to each well and the plate was incubated for 20 min. Then, 50 µL of stop solution was added to each well and the absorbance was quantified in a microplate reader (Bio-Rad Model 550). All samples and standards were measured in duplicate. In each experiment, a seven point standard curve was generated using 2-fold serial dilutions of recombinant human FAP from 4000 to 62.5 pg/mL in reagent diluent and a four parameter logistic curve-fit for each ELISA plate was constructed to calculate corresponding FAP concentrations in individual samples. The operator performing the ELISA was blinded regarding characteristics of all samples analyzed, and was not involved in data analysis. The authors were not involved in sample handling or laboratory analyses.

## Apparently healthy volunteers

We obtained EDTA plasma samples from 53 apparently healthy volunteers aged 18 to 87 years as control population. Individuals with acute illness, fever, chronic drug or alcohol abuse, neurological, cardiovascular, or malignant disease, recent immunizations, recent major or minor surgical treatments, acute or chronic infectious disease or possible contact with infectious individuals or material were excluded. All volunteers gave informed consent to blood sampling for research purposes. The study was approved by the ethics committee of Hannover Medical School (application number: 9362_BO_S_2020) and conforms to the ethical guidelines of the 1975 Declaration of Helsinki.

## Patients with Intra-Thoracic Cancer

We obtained EDTA-anticoagulated plasma blood samples from 18 patients with intra-thoracic malignancy (lung cancer, oesophageal cancer, lung metastatic cancer) who were admitted to the Department of Radiation Oncology, Hannover Medical School, for chest radiation therapy between December 2021 and April 2023. Blood sampling was performed in each patient at two time points, before the first radiation (baseline, *n* = 18) and after the last radiation session (post radiation, *n* = 16). Two patients declined blood sampling after finalization of radiation therapy. All patients gave informed consent to donation and blood sampling for research purposes. The study was approved by the ethics committee of Hannover Medical School (application number: 9362_BO_S_2020) and conforms to the ethical guidelines of the 1975 Declaration of Helsinki. Patient characteristics and radiation details of patients undergoing chest radiation are detailed in Supplementary Table S1. Plasma samples were frozen at -80 °C for measurement of FAP concentrations. Last radiation therapy ended in April 2024, and follow-up was accomplished for all patients by telephone contact with the patient, spouse, or general practitioner in June 2024.

We calculated the absolute change of FAP concentration (ΔFAP) in 16 patients. ΔFAP was calculated as difference of FAP concentration after radiation therapy vs. baseline. In two patients, post radiation plasma samples were not available, and therefore ΔFAP not calculated.

Routine laboratory analysis of leucocyte count, C-reactive protein (CRP), creatinine and calculated glomerular filtration rate (GFR) were performed at the Department of Clinical Chemistry at Hannover Medical School at baseline and post radiation in 8 patients (CRP: 7 patients). The authors were not involved in sample handling or laboratory analyses.

## Radiation therapy

Radiotherapy was administered to 18 patients, all receiving thoracic treatment. Treatment intention was (local) curative in 15 cases (83%) and palliative in 3 cases (17%). Indications included non-small cell lung carcinoma (NSCLC) (*n* = 8), esophageal carcinoma (*n* = 3), small cell lung carcinoma (SCLC) (*n* = 2) and lung metastases (*n* = 5) of various primary cancers, such as parotid carcinoma, rectal carcinoma, papillary carcinoma, sarcoma and urothelial carcinoma (*n* = 1 each). Moderate hypofractionated irradiation was used in 12 cases (67%) with single doses between 2 and 3 Gy. Cumulative treatment dose was median 50 Gy (25th – 75th percentiles 45–60 Gy). One patient received a stronger hypofractionated irradiation with single doses of 4 Gy, resulting in a cumulative dose of 60 Gy to the tumor core. Hypofractionated stereotactic radiotherapy (hfSRT) was used for lung tumors or pulmonary metastases in 5 cases (28%) [2].

In all cases image guided radiotherapy (IGRT) using modern volumetric modulated arc therapy (VMAT) was applied based on computed tomography radiation planning. Concomitant platin based chemotherapy was applied in 5 cases, either combined with etoposite (SCLC) or with paclitaxel (esophageal cancer) [23–25].

Treatment duration varied by diagnosis and therapy type (e.g., weeks for radiochemotherapy, days for stereotactic radiotherapy). We expected peak FAP changes at the end of radiotherapy.

Planning target volume size (PTV) was between 5 ml and 768 ml (median 177 ml, 25th -75 th percentiles 28 ml – 264 ml). Heart and (ipsilateral) lung were considered as relevant organs at risk (OAR) for this study. The mean cardiac dose was between 0.1 Gy and 10.7 Gy (median 0.7 Gy, 25th -75 th percentiles 0.3 –4.1 Gy). Mean lung dose (ipsilateral) was between 0.7 Gy and 12.4 Gy (median 8.7 Gy, 25th−75th percentiles 2.7–10 Gy) [26].

## Data Availability

All data supporting the findings of this study are available within the paper and Supplementary Data File 1.

### Statistical analyses

Continuous data are median with 25th and 75th percentiles. We used the Mann-Whitney-U-Test for comparison between two independent groups and Wilcoxon signed rank test for comparison of paired data. We used linear regression analysis to identify variables associated with plasma FAP and ΔFAP concentration, and the Kaplan-Meier method to illustrate patient survival during follow-up in relation to baseline concentrations of FAP. To evaluate the relationship between FAP and markers of kidney function and inflammation, we calculated the Spearman correlation coefficient. All analyses were performed with Statistica 8.0 (StatSoft) and Prism 5.0 (GraphPad).

## Results

### Circulating FAP concentrations in apparently healthy volunteers

A collection of 53 EDTA plasma samples taken from apparently healthy blood donors (18 men, 35 women, median age 30 years, age range 18–87 years) was assayed using the FAP ELISA. Volunteer blood samples had a median FAP concentration of 118 ng/mL (25th–75th percentiles 104–140 ng/mL, range 66–199 ng/mL). FAP concentrations in men (median 134 ng/mL, 25th–75th percentiles 111–172 ng/mL) were slightly higher than in women (median 114 ng/mL, 25th–75th percentiles 102–190 ng/mL, *P* = 0.02).

### Circulating FAP concentrations in Cancer patients

We measured FAP concentrations in plasma samples taken from patients before planned chest radiation for cancer therapy (*n* = 18, 12 men and 6 women) using the FAP ELISA. Baseline characteristics are presented in Suppl. Table S1.

**Fig. 1:**
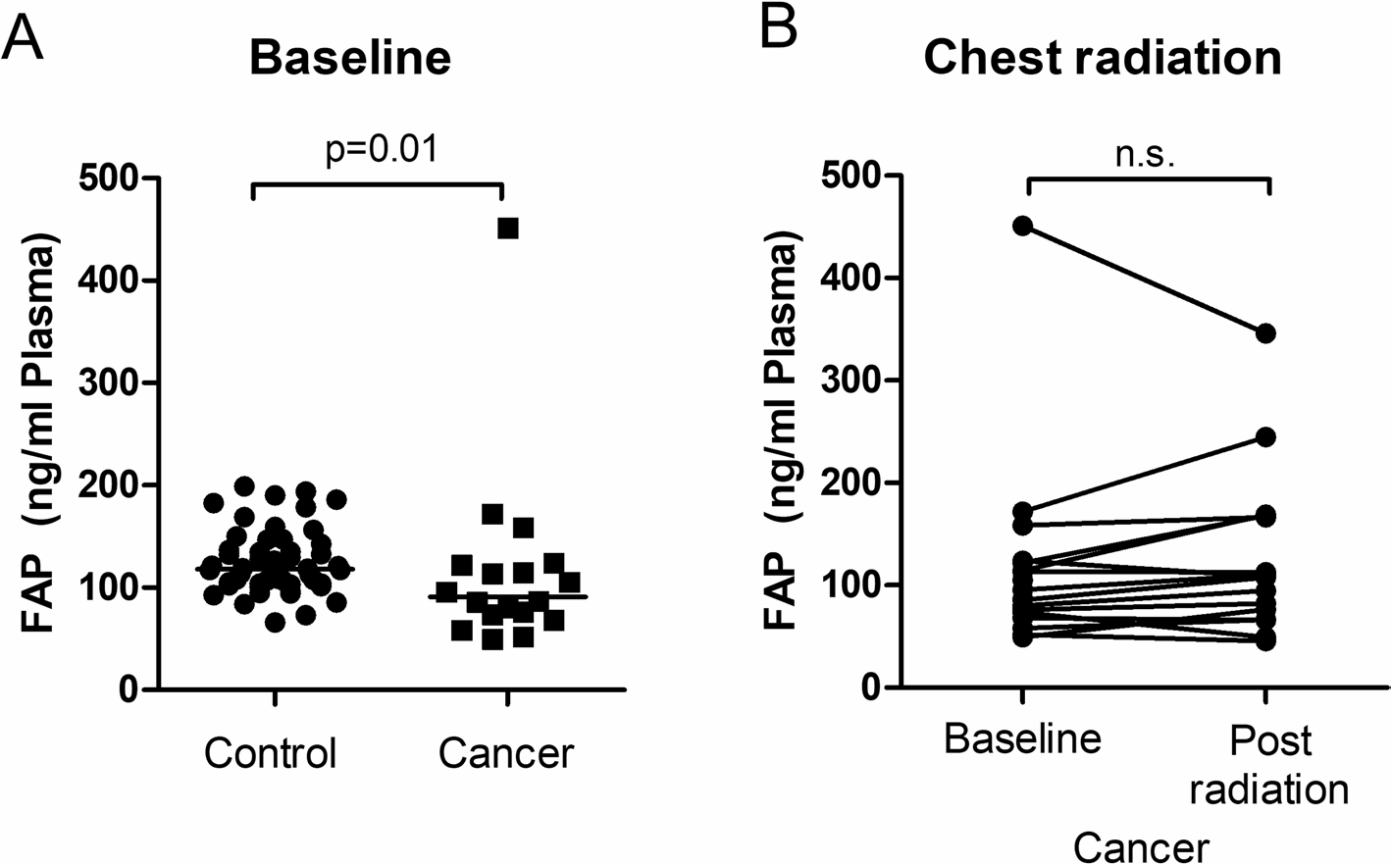
FAP Concentrations in Apparently Healthy Volunteers and Cancer Patients. **A** FAP concentrations in cancer patients are lower as compared to healthy volunteers (control). **B** In cancer patients, FAP concentrations are not different at baseline vs. post radiation therapy. **A**: control, *n* = 53; cancer, *n* = 18. **B**: cancer, *n* = 16

Blood samples had a median FAP concentration of 91 ng/mL (25th–75th percentiles 72–123 ng/mL, range 49–451 ng/mL), which was significantly lower as compared with healthy volunteers (*P* = 0.0002) (Fig. 1A). FAP concentrations in men (median 109 ng/mL, 25th–75th percentiles 75–149 ng/mL) were not different from those in women (median 81 ng/mL, 25th–75th percentiles 66–96 ng/mL, *P* = 0.28). Cancer patients (median age 67 years, age range 20–81 years) were older than apparently healthy volunteers (*P* = 0.0002 vs. volunteers). However, FAP concentrations were not related to age in healthy volunteers (r^2^ = 0.04, *P* = 0.14) or cancer patients (r^2^ = 0.04, *P* = 0.45).

Laboratory analysis in cancer patients revealed no change of C-reactive protein, leucocyte count, creatinine or GFR between baseline and post radiation therapy (Suppl. Figure 1). CRP-levels at baseline ranged from 1 to 159 mg/dL (median 46 mg/dL). One patient with NSCLC presented with elevated CRP and leucocyte levels at baseline and post radiation therapy; this patient had low FAP levels at baseline (58ng/ml) and post-radiation therapy (66ng/ml). However, no significant correlation of FAP levels with CRP or any other laboratory parameter was found in the cancer patient cohort (Suppl. Table 3). There was a positive correlation between CRP and leucocyte count (0.93, *p* = 0.007) and a negative correlation between Creatinine and GFR (− 0.76, *p* = 0.037).

### Circulating FAP concentrations in response to chest radiation

To analyze changes of FAP concentrations due to radiation effects, we measured FAP concentrations in plasma samples from the same cancer patients after finalization of chest radiation therapy. Post-radiation FAP concentrations (median 108 ng/mL, 25th–75th percentiles 71–167 ng/mL, range 46–346 ng/mL) were not different to baseline FAP concentrations (*P* = 0.19, Fig. 1B). FAP concentrations increased in 10 (63%) and decreased in 6 cancer patients (38%), indicating variable FAP homeostasis in individual patients. Time between baseline and final blood sampling was 31 days (median, range 9–46 days).

To better understand the change of FAP concentrations after chest radiation, we calculated the absolute amount of FAP change from baseline to final blood sampling as delta FAP (ΔFAP) for 16 patients. After chest radiation, median ΔFAP was 8 ng/mL (25th–75th percentiles − 4 to 25 ng/mL, range − 105 to +73 ng/mL). ΔFAP values were not different in men (median + 14 ng/mL, 25th–75th percentiles − 6 to +46 ng/mL) and women (median + 6 ng/mL, 25th–75th percentiles − 9 to +15 ng/mL) (*P* = 0.50).

### Circulating FAP concentrations are independent of Cancer type and radiation therapy

**Fig. 2:**
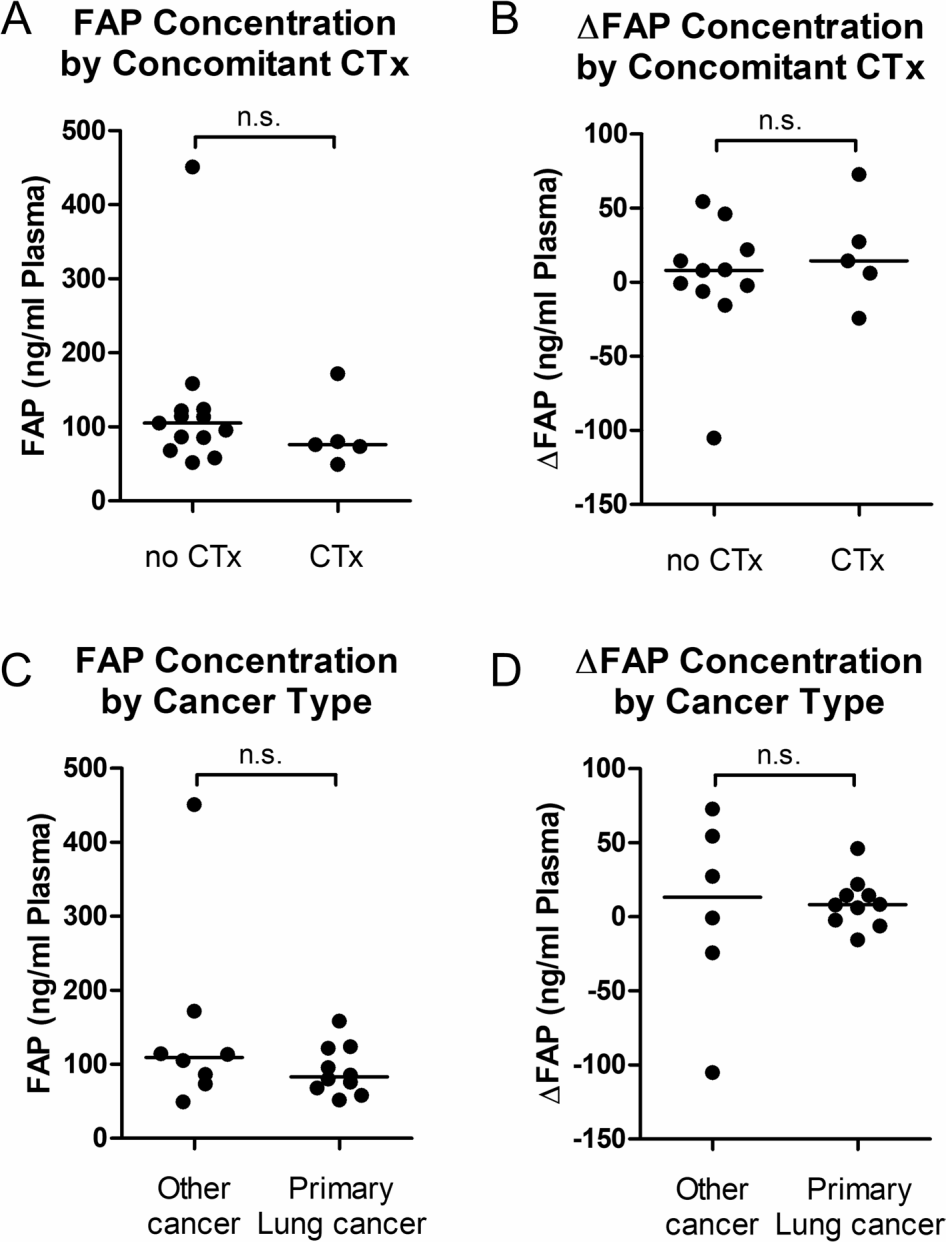
FAP concentrations are independent of chemotherapy or cancer type. **A**,** B** Baseline FAP concentration and change of FAP concentration (ΔFAP) are independent of concomitant chemotherapy. (**C**,** D**) Baseline FAP concentration and ΔFAP are independent of cancer type (primary lung cancer: SCLC, NSCLC; other cancer: esophageal, lung metastatic cancer). **A**, **C**: *n* = 18. **B**, **D**: *n* = 16

FAP concentrations before and after radiation therapy as well as ΔFAP were independent of cancer type and not different in patients with or without concomitant chemotherapy (Fig. 2A–D). By linear regression analysis, FAP concentrations before and after radiation as well as ΔFAP were not correlated with time between blood sampling, cumulative radiation dose, organ target volume, heart and lung organ dose (Supplementary Table 2). There was a significant correlation of FAP concentrations before and after radiation and ΔFAP with the highest single radiation dose. However, this was due to only one patient with metastatic prostate cancer demonstrating very high FAP concentrations (baseline: 451 ng/ml; post radiation: 346 ng/ml) who received a single dose of 18 Gy and a cumulative radiation dose of 54 Gy. Linear correlation diminished when the outlier patient was excluded from the analysis.

### Outcome in relation to FAP concentrations

Six patients (33%) died during follow-up. Median time to death was 3 months after first radiation therapy (range 1–16 months). The remaining patients survived until end of follow up (median 20 months, range 15–30 months).

**Fig. 3:**
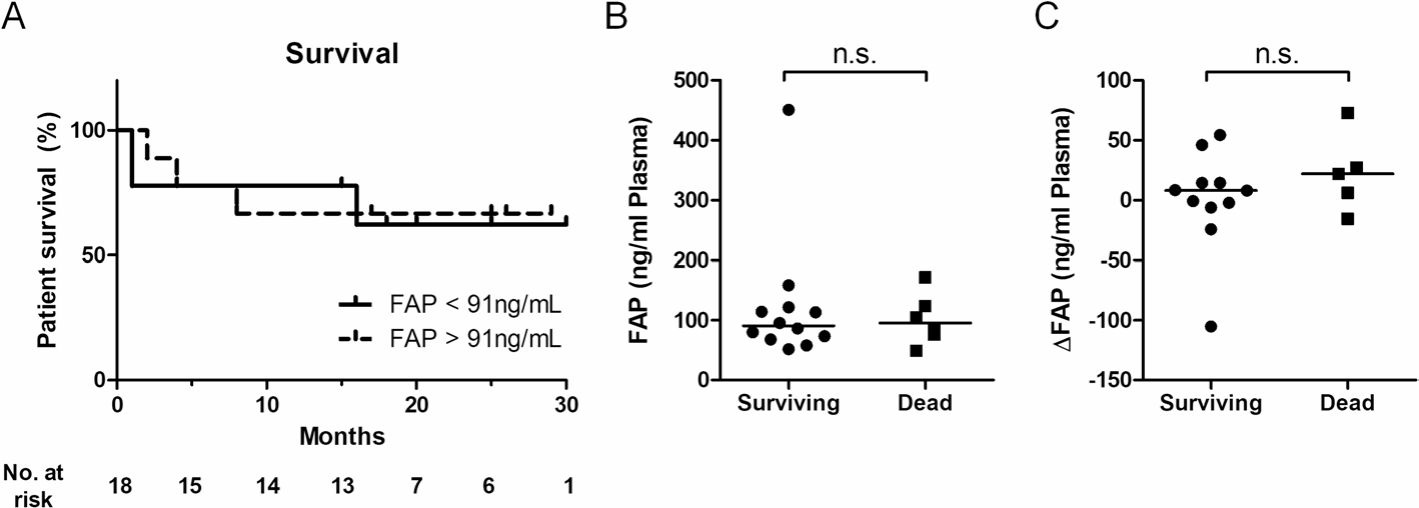
FAP concentrations in cancer patients are not related to survival. **A** When dichotomized by median baseline FAP concentration (91 ng/mL), survival of cancer patients is not different between patients with low or high baseline FAP concentrations. **B** FAP concentrations and change of FAP concentration (ΔFAP) are not different between surviving and deceased cancer patients. **A**, **B**: *n* = 18; **C**: *n* = 16

There was no difference in mortality between patients presenting with FAP concentrations below or above median before radiation therapy (cutoff value 91ng/mL, Fig. 3A). Moreover, there were no differences in absolute FAP concentrations and ΔFAP between surviving and deceased patients (Fig. 3B, C).

## Discussion

### Circulating FAP concentrations in apparently healthy volunteers and Cancer patients

In this study, we investigated the potential effects of chest radiation therapy on circulating FAP concentrations. The FAP concentrations in healthy volunteers were consistent with previous findings in healthy blood donors [27]. However, cancer patients demonstrated significantly lower median FAP concentrations before radiation therapy as compared to healthy volunteers. This finding reconciles with previous studies reporting low circulating FAP concentrations in various malignancies: In a large study with 561 patients newly diagnosed with malignancies including head and neck, gastrointestinal and genitourinary tumors, circulating FAP concentration was lower in cancer patients as compared to healthy volunteers [22]. Similar results were shown in renal cell carcinoma [28] and colorectal adeno-carcinoma [14]. Low circulating FAP concentrations were associated with higher tumor stage in patients with esophageal squamous cell carcinoma [29].

Until now, the physiological and pathophysiological homeostasis of circulating FAP is poorly understood. Alterations in circulating FAP concentrations may result from tumor-associated changes in fibroblast activity or systemic protease activity, which could modulate FAP production or clearance. We previously demonstrated that soluble FAP is regulated by serine and matrix metalloproteinases [10]. Hence, reduced FAP concentrations in cancer patients might reflect diminished release of FAP from tumor-associated fibroblasts or increased consumption of FAP within the tumor microenvironment. Still, there might be other yet unknown factors regulating FAP concentrations.

### Circulating FAP concentrations in response to chest radiation

In our study, circulating FAP concentrations did not change between initial and final radiation therapy. This could be due to the relatively short interval of 31 days (median) between the two blood samplings. However, it has been demonstrated previously that chest radiation induces pulmonary inflammation as early as two weeks post treatment, in part mediated by TGFβ_1_ [5]. Moreover, alterations of lung epithelial cell phenotype have been detected within a few days to 28 weeks after radiation, reflected by changes in pulmonary and circulating lung epithelial cell derived club cell secretory protein [6]. Still, FAP concentrations might change at a later time after radiation and remained undetected in our study.

We observed variability in FAP responses, with increases in 10 patients and decreases in 6 patients suggesting individualized pathophysiological responses to radiation. Therefore, other factors may have influenced FAP homeostasis in cancer patients studied: Groves et al. showed inter-individual variability in radiation sensitivity using an experimental radiation lung injury model in mice [6]. Additionally, the lack of differences in circulating FAP concentrations across cancer types and chemotherapy status suggests that other, yet unknown factors might regulate FAP homeostasis in these patients.

We have previously shown that current smoking and elevated CRP levels are associated with lower FAP concentrations in patients with acute coronary syndromes [27]. In the present study, one patient with high CRP and leukocyte levels, indicative of ongoing inflammation, exhibited low FAP concentrations. However, we found no significant correlation between FAP and CRP or leukocytes. This lack of correlation may be attributed to the small sample size but also by the fact that smoking and CRP levels may account only for a minor portion of the variability in plasma FAP concentrations as has been shown before [27].

Cancer patients in our study were older than controls and had comorbidities such as hypertension, diabetes or coronary artery disease, which could have influenced FAP levels. However, our previous research demonstrated that FAP values were not dependent on these factors. Moreover, FAP levels in patients with stable coronary artery disease did not differ from those measured in healthy blood donors this report [27].

Another explanation is that local radiation effects such as inflammation are not reflected in plasma concentrations of FAP, as has been shown by our group before in cardiovascular disease: Circulating FAP concentrations in patients with acute myocardial infarction or aortic stenosis were independent of localized FAP expression in the heart as analyzed by whole body nuclear imaging using positron emission tomography [10].

### Outcome in relation to FAP concentrations

In our study, baseline and post-radiation FAP concentrations did not predict mortality in cancer patients undergoing radiation therapy. This is in contrast to our previous findings in patients with cardiovascular disease, where low FAP concentrations were linked to worse outcomes in patients with acute coronary syndrome [27] and stroke [21]. The lack of a clear relationship between FAP concentrations and survival in cancer patients in our study suggests that early post-radiation FAP measurements may not predict cancer outcomes or radiation response. Mortality in these patients is also influenced by the underlying diagnosis and treatment intent, as survival is generally longer in patients receiving curative radiotherapy compared to those undergoing palliative treatment. Given this variability our sample size may not have been large enough to detect outcomes and further studies with larger cohorts are needed to clarify this potential relationship.

### Study limitations

A limitation of this study is the small sample size which restricts the power of our findings. Additionally, while chronic radiation-induced pulmonary fibrosis is rare, acute inflammatory injury is common. Our measurements may have missed potential FAP changes occurring over longer post-radiation intervals. Future studies with larger cohorts and extended follow-up are necessary to confirm our findings and evaluate the utility of circulating FAP as a biomarker in cancer patients post-radiation.

## Conclusion

This study is the first to analyze circulating FAP concentrations in patients with intra-thoracic cancer undergoing radiation therapy. We demonstrate that cancer patients have lower FAP concentrations as compared to healthy volunteers. However, baseline FAP and post-radiation FAP concentrations do not appear to be suitable as biomarkers for radiation-induced fibroblast activation. Further studies are warranted to elucidate the mechanisms underlying reduced FAP concentrations in cancer patients and assess its potential as a biomarker.

## Electronic supplementary material

Below is the link to the electronic supplementary material.

Supplementary Material 1

Supplementary Material 2

## Data Availability

All data supporting the findings of this study are available within the paper and Supplementary Data File 1.

## Abbreviations

FAP: Fibroblast activation protein alpha
PTV: Planning target volume
OAR: Organs at risk
IGRT: Image-guided radiotherapy
VMAT: Volumetric modulated Arc therapy
