# Supplementary material for: Circulating soluble fibroblast activation protein (FAP) in patients with intra-thoracic cancer undergoing chest radiation
Source: Discov Oncol. 2025 Jul 21;16:1381. doi: 10.1007/s12672-025-02998-y (PMC12279648; doi:10.1007/s12672-025-02998-y)
Supplement: Supplementary file 1 — Supplementary Material 1 [file 12672_2025_2998_MOESM1_ESM.docx]

**Tables**

Supplementary Table 1: Baseline characteristics and radiation details of cancer patients undergoing chest radiation

| n | 18 |  |
| --- | --- | --- |
| Age, years | 67 (62-75) |  |
| Male sex | 12 (67) |  |
| Comorbidities present | 16 (90) |  |
| Multiple Comorbidities present | 14 (78) |  |
| Coronary artery disease | 9 (50) |  |
| Diabetes | 4 (22) |  |
| Myocardial infarction | 4 (22) |  |
| Chronic inflammatory diseases | 4 (22) |  |
| Hypertension | 3 (17) |  |
| Chronic kidney disease | 3 (17) |  |
| Alcoholism | 2 (11) |  |
| Smoking | 1 (6) |  |
| Glaucoma | 1 (6) |  |
| Thyroid disease | 1 (6) |  |
| Creatinine, mmol/L | 69 (63-111) |  |
| GFR, mL/min | 88 (66-90) |  |
| CRP, mg/L | 46 (1-126) |  |
| Leucocytes, x10³ | 7.4 (4.6-9.8) |  |
| Concomitant chemotherapy | 5 (28) |  |
| Radiation duration (fractions) | 15 (9-24) |  |
| Radiation dose, cumulative (highest, Gy) | 50 (45-60) |  |
| Radiation dose, single (highest, Gy) | 3 (2-9) |  |
| Planning Target Volume, PTV (ml) | 177 (28-264) |  |
| Organ-dose: Heart (mean, Gy) | 0.7 (0.3-4.1) |  |
| Organ-dose: Lung (mean, Gy) | 8.7 (2.7-10) |  |

Data are n (%) or median (25th–75th percentile).

CRP: n=7; Creatinine, GFR, Leucocytes: n=8

Supplementary Table 2: Linear regression analysis of FAP concentrations in cancer patients undergoing chest radiation

|  | FAP  baseline | |  | FAP  post radiation | |  | ΔFAP | |
| --- | --- | --- | --- | --- | --- | --- | --- | --- |
|  | r² | P |  | r² | P |  | r² | P |
| Radiation duration (days) | 0.03 | 0.49 |  | 0.02 | 0.64 |  | 0.04 | 0.46 |
| Radiation dose, cumulative | 0.00 | 0.82 |  | 0.03 | 0.49 |  | 0.02 | 0.57 |
| Radiation dose, single | 0.50 | 0.001 |  | 0.32 | 0.02 |  | 0.56 | 0.001 |
| Target volume | 0.15 | 0.11 |  | 0.22 | 0.07 |  | 0.01 | 0.78 |
| Organ-dose: Heart | 0.19 | 0.08 |  | 0.22 | 0.08 |  | 0.10 | 0.24 |
| Organ-dose: Lung | 0.08 | 0.27 |  | 0.06 | 0.34 |  | 0.07 | 0.33 |

FAP baseline: n = 18; FAP post radiation, ΔFAP: n=16

Supplementary Table 3: Correlation analysis of FAP and blood markers of kidney function and inflammation at baseline

|  | Correlation coefficients | | | | | P values | | | | |  |
| --- | --- | --- | --- | --- | --- | --- | --- | --- | --- | --- | --- |
|  | | FAP | CRP | Creatinine | GFR | Leuco-cytes | FAP | CRP | Creatinine | GFR | Leuco-cytes |
| FAP | |  | -0.43 | 0.29 | -0.41 | -0.55 |  | 0.354 | 0.501 | 0.327 | 0.171 |
| CRP | | -0.43 |  | -0.46 | 0.36 | 0.93* | 0.354 |  | 0.302 | 0.444 | 0.007 |
| Crea | | 0.29 | -0.46 |  | -0.76* | -0.36 | 0.501 | 0.302 |  | 0.037 | 0.389 |
| GFR | | -0.41 | 0.36 | -0.76* |  | 0.29 | 0.327 | 0.444 | 0.037 |  | 0.501 |
| Leuco-cytes | | -0.55 | 0.93* | -0.36 | 0.29 |  | 0.171 | 0.007 | 0.389 | 0.501 |  |

FAP, Creatinine, GFR, Leucocytes: n = 8; CRP: n=7. Significant correlation coefficients are marked (asterisk)

Supplementary Figure 1:

Supplementary Figure 1: Blood markers of kidney function and inflammation at baseline and after radiation therapy.

Serial measurements in patients undergoing radiation therapy revealed no change of C-reactive protein (CRP), Leucocyte count, Creatinine or glomerular filtration rate (GFR) between baseline and post radiation therapy. One patient with NSCLC presented with elevated CRP and leucocyte levels at baseline and post radiation therapy (asterisk). This patient had low FAP levels (58ng/ml at baseline).

Black circles: Concomitant chemotherapy; white squares: no chemotherapy; CRP, n=6; Leucocytes, Creatinine, GFR, n=7.
